# Secretion pattern of canine amniotic stem cells derived extracellular vesicles

**DOI:** 10.1590/1984-3143-AR2022-0063

**Published:** 2022-11-14

**Authors:** Rafael Garcia Karam, Lina Castelo Branco Motta, Matheus Ferreira de Almeida, Alessandra Bridi, Juliano Coelho da Silveira, Carlos Eduardo Ambrósio

**Affiliations:** 1 Departamento de Cirurgia, Faculdade de Zootecnia e Engenharia de Alimentos, Universidade de São Paulo, Pirassununga, SP, Brasil; 2 Departamento de Medicina Veterinária, Faculdade de Zootecnia e Engenharia de Alimentos, Universidade de São Paulo, Pirassununga, SP, Brasil

**Keywords:** extracellular vesicles, canine, mesenchymal

## Abstract

Extracellular vesicles (EVs) derived from stem cells (SCs) have regenerative potential and the possibility of being used in treating chronic diseases. EVs present lower risk of tumorigenicity and easily to isolation and storage. Therefore, this research aims to compare the morphological characteristics of the EVs (up to 150nm) derived from stem cells obtained from canine amniotic membranes in different passages during the *in vitro* culture. For this, cells from the amniotic membranes were isolated, cultured, and characterized. In order to answer our aim, the number of cells was normalized at each passage to generate conditioned media for EVs separation. The cells were differentiated into adipogenic, chondrogenic, and osteogenic tissue, to characterize these cells as mesenchymal stem cells (MSC). Moreover, flow cytometry analysis was performed and showed that the MSC were positive for CD90, CD105 and negative for CD34, CD45, mesenchymal and hematopoietic markers, respectively. For EVs analysis, MSC in different passages (P0-P2) were culture until 80% of confluence, then the medium was replaced by EVs depleted medium. After 48h, culture medium was collected and centrifuged to separate EVs, followed by nanoparticle tracking analysis. The EVs were also characterized by western blot and transmission electron microscopy (TEM). EVs were positive for Alix and negative for Cytochrome C as well as presented the traditional cup-shape by transmission electronic microscopy. Our results demonstrated that the concentration in the different passages was increased in P0 compared to P1 and P2 (p<0.05). No differences were found in EVs size (P0=132nm, P1=130nm and P2=120nm). Together, these results demonstrate that P0 of MSC is enriched of EVs when compared to later passages, suggesting that this passage would be the best to be applied in pre-clinical tests. Despite that, more studies are necessary to identify the EVs content and how the cells will respond to treatment with them.

## Introduction

With the increase in the number of chronic diseases in the world, the search for alternative and innovative therapies has been growing gradually. Since the discovery of the therapeutic potential of stem cells (SC) in tissue regeneration, its studies have continued to grow in order to solve problems caused by various pathologies. However, its use has been generating some ethical and moral problems due to the use of cells from embryonic origin, a fact that is not always acceptable by most people. For their use in veterinary medicine, SC are extracted from several sources of fetal or adult tissue, and among them is the amniotic membrane, which has low tumorigenicity and greater multipotentiality when compared to other adult tissues, which makes them more accessible for allogeneic transplants (Cardoso et al., 2017).

In order to increase our understanding about mesenchymal stem cells derived from the canine amniotic membrane (MAC), we also need to study the components secreted by these cells, such as extracellular vesicles (EVs). These vesicles can be classified according to size, (<200nm) small or (> 200nm) large (Théry et al., 2018). Extracellular vesicles are the result of a complex process of cell secretion and contain a lipid bilayer and spherical shape, in addition to its interior containing proteins, bioactive lipids, microRNAs (miRNAs) and mRNAs (Zhang et al., 2015; Bonafede et al., 2016; Huang et al., 2016; The secretion of these vesicles results in a cellular interaction, which can be carried by body fluids to any part of the body, making this communication virtually unrestricted.

Based on the above information it is important to understand the role of extracellular vesicles in stem cells dynamics in vitro. Stem cells isolation initiate with a primary cell culture and through the cell culture passages a pure population is separated and multiplied for differ purposes. Extracellular vesicles could be involved in cell proliferation as well as differentiation. Based on that this work aims to compare the quantity and size of small extracellular vesicles (up to 200nm) derived from canine amniotic membrane stem cells throughout different passages in vitro.

## Methods

This study was submitted to the animal ethics committee of the Faculty of Veterinary Medicine and Zootechnics of the University of São Paulo - FMVZ/USP, and its performance was approved (CEUA nº 2486170118).

Fetuses of gestational age over ±42 days (third-final gestation) were collected, using the methodology proposed by Evans; Sack, (1973) and Pieri et al. (2015). Briefly, the amniotic membrane was mechanically separated from the rest of the fetus and the samples were obtained as described by Park et al. (2012). MAC was subjected to enzymatic digestion using collagenase type I (1-2 mg/ml; C2674-100MG, SIGMA-ALDRICH) and incubated at 38 °C, with a relative humidity of 80% and 5% CO_2_, for approximately two hours. Subsequently, the tissue samples were inactivated with 1 ml of DMEM/F12 medium (Invitrogen, cat. No. 10565-018) and centrifuged at 300 g for 5 minutes. The supernatant was discarded, and the pellet resuspended in 1 ml of DMEM/F12 medium, supplemented with 10% fetal bovine serum (SFB - Invitrogen. Cat. No. 12657-029), 1% non-essential amino acids (Sigma. Cat. No. M-7145), 1% L-glutamine (Life. Cat. No. 25030-081), 1% penicillin/streptomycin and incubated at 38 °C.

Next, cells were subjected to a growth curve characterization as described by Vidane et al. (2014). Cells counting was done by removing the medium and adding 2 mL of trypsin for approximately 10 minutes at 38.5 °C. After this process, the solutions were transferred to a 15 mL tube and an additional 2 mL of culture medium was added to inactivate trypsin. The tubes were centrifuged for five minutes at 300 xg. A pellet was formed, and the solution discarded. A total of 1 ml of culture medium was added and the pellet was resuspended to homogenate the sample. To achieve the cell number 10 µL of the solution was placed in a Neubauer Chamber for cell counting.

The ability of canine amniotic membrane stem cells to form colonies was assessed according to the protocol of Vidane et al. (2014) and Ercolin et al. (2016). In a 100 mm diameter culture plate, cells were plated at densities of 1x10^3^, 1x10^4^ and 1x10^5^ in the first pass and incubated at 38 °C in an atmosphere of 5% CO2. The culture medium was replaced every four days, for approximately 13 days. After this period, the cultures were washed twice with PBS 1X buffer solution, fixed in 4% paraformaldehyde for 30 minutes, washed again with PBS buffer and stained with 0.1% Giemsa for 15 minutes at room temperature. After the last step, the number of cell colonies was observed and counted.

In order to characterize, cells were differentiated in vitro into adipogenic, chondrogenic and osteogenic lines, according to the protocol of Vidane et al. (2014). Flow cytometry was also performed for cell characterization. Cells were distributed in microtubes with the addition of 50 µL of PBS with 10% rabbit blood serum for 20 minutes. After this period, the primary antibody was added for 20 minutes at room temperature (CD105 dilution pure, IgG2b, catalogue number 156,756, Abcam; CD34 1:100, IgG1, catalogue number 12-0340-42, eBioscience; CD90 1:100, IgG2b, catalogue number 12-5900-42, eBioscience; CD45 1:100, IgG2b, catalogue number 11-5450-42, eBioscience).Then, cells were centrifuged, and the secondary antibody was added for 20 minutes at 4 °C (1:50; Goat anti-mouse IgG, catalogue number F0479, Company Dako) After this period, cells were washed and centrifuged. The content was distributed in specific Falcon tubes and analyzed in a flow cytometer using a FACSCAria Cell Sorter software V.6.1.2 (BD Biosciences, San Jose, CA, USA). The filters used in the above equipment were adjusted for the emission light for the fluorochrome FITC (525 nm).

### EVs analysis

To isolate EVs from canine amniotic cells, from four different fetuses were previously cultured, differential centrifugation and ultracentrifugation steps were used, using their conditioned culture medium. When cells in culture reached 80% confluence in the passage P0, P1 and P2, the culture medium was exchanged for another EVs-free medium, which had previously been centrifuged (119700 xg for 14 hours to remove EVs from medium), the new medium remained together with the cells for 48 hours and then removed for EVs analyses.

Cell culture media from each passage were subjected to consecutive centrifugations, the first being for 300 xg for 10 min, where after the process the pellet containing cells was discarded and the supernatant centrifuged again at 2,000 xg for 10 minutes, the pellet containing cell debris was again discarded and the supernatant containing EVs was centrifuged at 16,500 xg for 30 min, both at 4 °C (Gyrozen, 1730R). The supernatant containing EVs were stored in a freezer at -80 °C for further analysis

After thawing the previously stored supernatant at room temperature, they were subjected to a 0.22 µm syringe filter (KASVI K18-230) and weighed to maintain the same mass, due to the high sensitivity of the ultracentrifuge (Optima XE- 90 Ultracentrifuge). Samples were subjected to 119,700 xg for 1:10 hours at 4 °C. Then the supernatant was removed and resuspended 2 ml of PBS and centrifuged again under the same conditions. At the end of the second centrifugation, the EVs were resuspended in 20µL of PBS for further analysis.

### Nanoparticle tracking analysis

Nanoparticle tracking analysis was performed to evaluate size and concentration of extracellular vesicles that were previously separated from stem cells conditioned culture medium. Cell culture medium was collected in P0, P1 and P2, all containing the same number of cells, in order to compare their EVs size and concentration.

To characterize the EVs, Nanosight (NS300, NTA 3.1 Build 3.1.45; Malvern) was used following protocols previously used (Hung and Leonard, 2016). The videos were made with the camera (sCMOS) at level 14 at 37.0 ° C, calibrating with 100 nm of beads to ensure the accuracy of the equipment. The EVs were diluted in PBS (1: 100) and placed in the nanosight (500µL), for photo documentation. A total of five videos of 30 seconds each were made and considered the threshold 5 for data analysis.

### Protein marker analysis

Westerns Blotting was performed to characterize EVs with specific antibodies used to identify small extracellular vesicles as well as markers for cell contamination. The small EVs were isolated and resuspended in RIPA (Sigma-Aldrich, St. Louis, Missouri, USA), as well as the stem cells from which they were derived for control, the protocol was used according to the manufacturer instructions. Samples were transferred to the gel containing 12% SDS-PAGE polyacrylamide (Bio-Rad, Hercules, California, USA). For a period of 2:30 hours, the samples ran at 100 volts and then the gel was placed together with the nitrocellulose membrane (Biotrace NT, Pall Life Sciences, Pensacolla, Florida, USA), followed by a wet transfer for 120 minutes at 80 volts. After this procedure, the membrane was incubated in a blocking solution containing 5% BSA with tris buffered saline with Tween 20 for one hour. Subsequently, the primary rabbit polyclonal anti-Alix antibody (0.5 µg/mL, N-20: SC-49267, Santa Cruz Biotechnology), ACTB (0.5 µg/mL, SC-15363, Santa Cruz Biotechnology) and Cytochrome C (C-20: SC-8385, Santa Cruz Biotechnology) were added and incubated for at least 12 hours at 4 °C. Then the membrane was washed three times for five minutes in 0.1% Tween 20 saline tris buffer solution, followed by one hour incubation with the secondary anti-rabbit IgG antibody (HRP-linked Antibody 7074S) for Alix and Donkey anti -goat (IgG-HRP SC-2020) for Cytochrome C. Afterwards three washes of the membranes were started with five minutes each with 1X of 0.1% tris saline buffer and the ECL Plus Prime Western Blotting Detection System Solution was added (Amersham, Buckinghamshire, UK) for further development and image capture of the bands by ChemiDoc MP Image System (Bio-Rad, Hercules, California, USA).

### Transmission electron microscopy

Extracellular vesicles pellets isolated from 2,500 µl of the canine amniotic stem cells conditioned medium, were diluted in 200μl of fixing solution (1% cacodylate; 2% glutaraldehyde and 2% paraformaldehyde at pH 7.2-7.4) and maintained for 2h at room temperature. After, EVs were diluted in 2 mL of 1X Ca^2+^/Mg^2+^ free PBS, and the solution was centrifuged once in order to obtain fixed EVs pellet (119,700 xg, 70 min, 4 °C). The pellet was diluted in 20μL of buffer solution (1% cacodylate) and kept refrigerated until analysis. The EVs solution was placed in a copper grid for 50 min at room temperature in order to dry before staining. In the grid it was added 2% of uranyl acetate and performed the analysis of EVs morphology using a transmission electron microscope (FEI Tecnai 20; LAB6 emission; 200kV).

### Statistical analysis

The quantification and size analysis of EVs were analyzed by the comparison of variances (ANOVA) test followed by Tukey comparative test. The level of significance considered in all tests was p ≤ 0.05. All analyzes were performed using the JMP 7 program.

## Results

### Canine amniotic cells collection and processing

**Figure 1:**
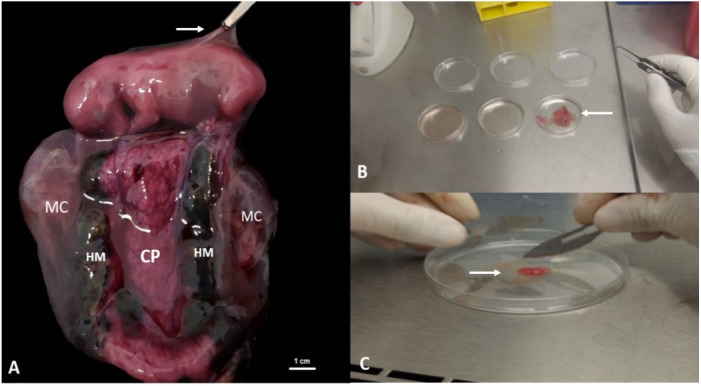
Sample collection and processing. In (A) canine fetus after removal from the uterine horn. In (B) canine amniotic membrane being cleaned in different plates containing PBS with 5% penicillin/streptomycin.

The use of pregnant uteri from bitches submitted to ovarian hysterectomy in this study proved to be efficient for obtaining MAC. The material was carefully collected and dissected with the aid of a delicate surgical instrument, until only the fetus involved in MAC was left. The amniotic membrane was removed, giving preference to the use of the dorsal cover of the fetus, avoiding contact with hematopoietic cells of the umbilical cord (Figure 1).

**Figure 2:**
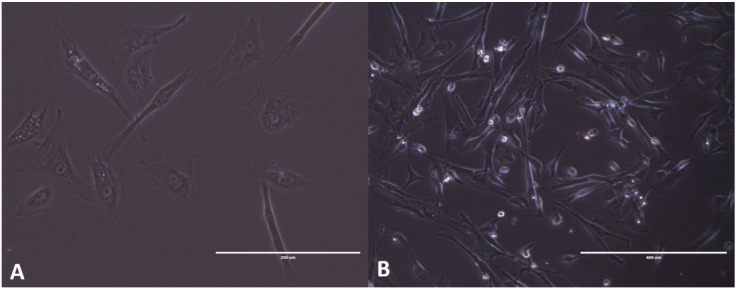
Cell morphology upon culture. In (A) cells still in P0, after 48 hours of cell culture, the cells showed adhesion to the plastic of the culture bottle and with a fibroblast aspect. In (B) after its transition to P1 with high confluence and ready to be treated with trypsin again.

Cleaning the material with PBS solution and supplemented with 5% penicillin/streptomycin proved to be efficient, thus avoiding contamination during the following steps. The use of collagenase type I showed a variable time of two to three hours to digest the MAC, requiring homogenization of the sample every 30 minutes to improve the process. After the process of maceration and enzymatic digestion, cells were grown under controlled conditions described above, and their growth verified every 24 hours. When the cells had 80% confluence, they were removed with trypsin and cultured again in additional passages for their expansion and conservation to a MAC cell bank (Figure 2).

In (C) canine amniotic membrane being macerated with the aid of a scalpel blade. (MC) chorioallantoic membrane, (CP) placental brace, (HM) marginal hematoma, (white arrow) amniotic membrane.

**Figure 3:**
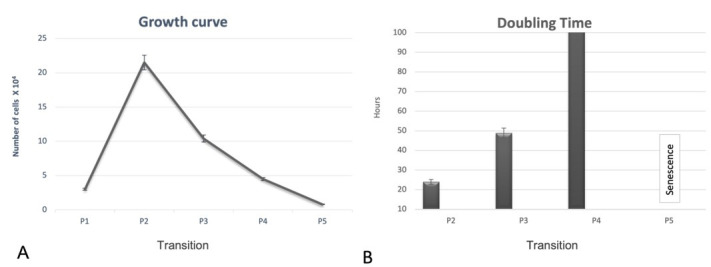
Growth curve and cell numbers. In (A) Graph showing the doubling time of stem cells derived from the canine amniotic membrane. Note that in the fifth pass (P5) the cells entered into cell apoptosis. In (B) Graph showing the doubling time of stem cells derived from the canine amniotic membrane. Note that in the fifth pass (P5) the cells entered into cell apoptosis. Standard bars represent the standard error of the mean (SEM).

After the cryopreservation process, the cells were observed for their viability. The cells showed high post-freezing cell viability, close to 90%. The growth curve pattern was possible to be established until the fifth passage, due to the number of cells counted at the end being less than the number of cells plated. In the second passage (P2) it was possible to notice a peak of growth and a gradual reduction in proliferation until the fifth pass. In the Doubling time it was possible to observe a considerable increase in the number of days required for cell duplication, that is, there is a gradual decline in the number of cells as the number of passages increases (Figure 3).

### Stem cell lineage characterization

**Figure 4:**
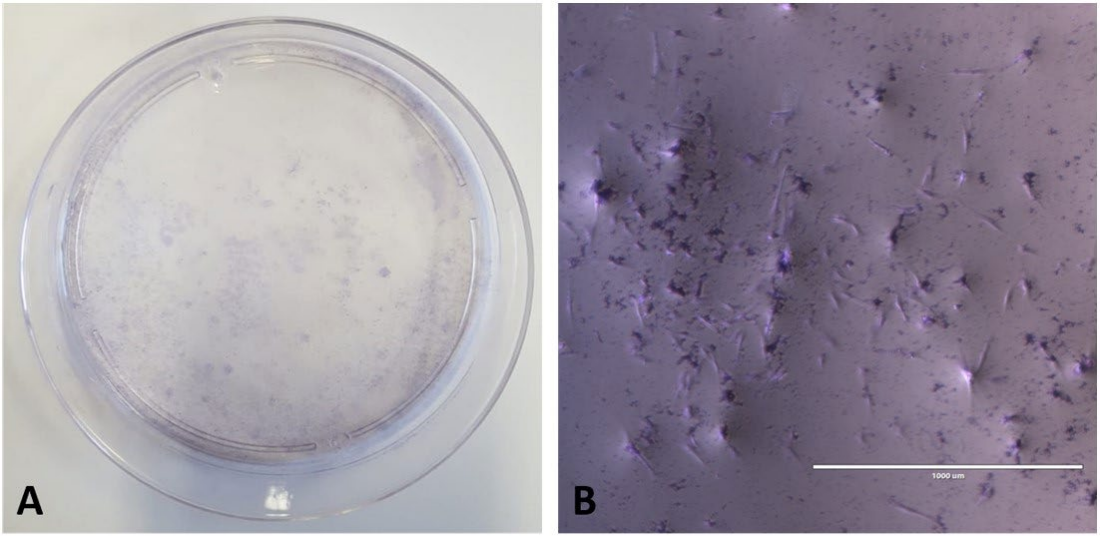
Colony formation analysis. Colony-forming Unit Test (CFU) of canine amniotic membrane cells with Giemsa. In (A) plate with CFU. In (B) photomicrograph of CFU in 4x magnification (Scale bar: 1000µm).

Colony-forming unity (CFU) was performed and a density of 1x10^4^ was established, with colony formation after 13 days. After staining, it was possible to observe clustered and purple-colored cells (Figure 4).

**Figure 5:**
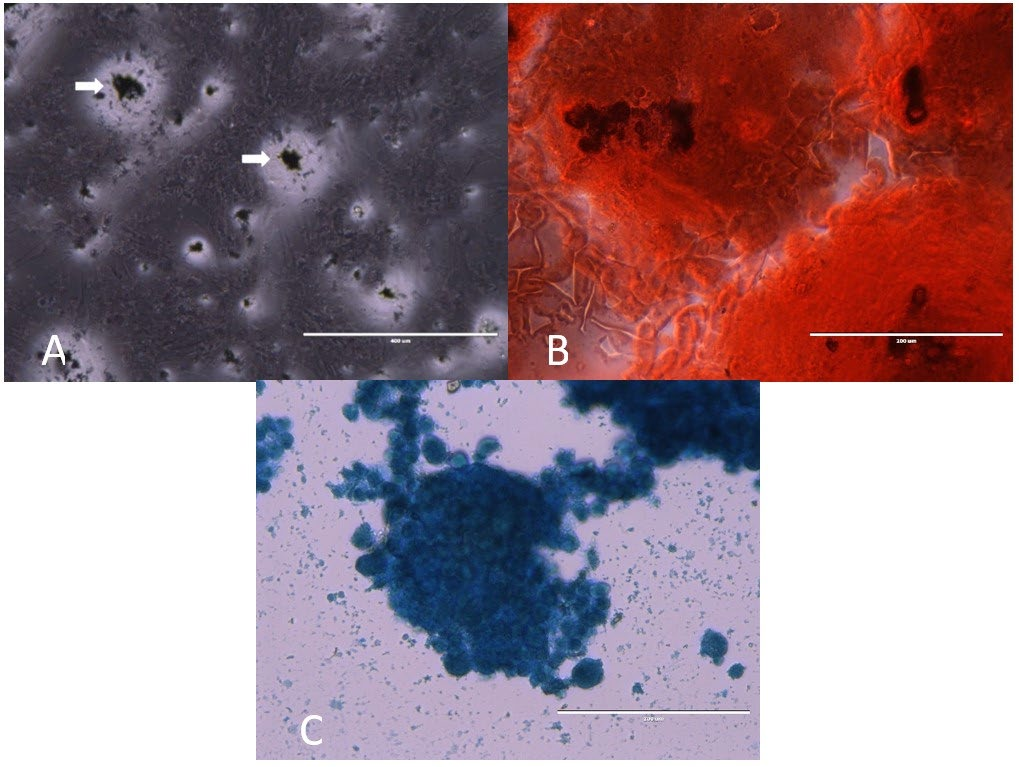
Cell differentiation analysis. In (A) Photomicrograph of MAC cells after differentiation in adipogenic. In (B) chondrogenic and in (C) osteogenic media. 40X increase (Scale bar: 400µm).

After adipogenic differentiation, it was possible to notice the appearance of innumerable fat droplets, and as the cultivation increased, the droplets also multiplied. Upon Sudan-Black staining it was possible to determine the presence of lipid droplets in black. Additionally, we demonstrated the chondrogenic differentiation of MAC cells, based on collagen blue staining. MAC cells were cultured in an osteogenic differentiating medium. The results demonstrated the deposition of extracellular amorphous mineral material, staining the entire plate in red (Figure 5)

**Figure 6:**
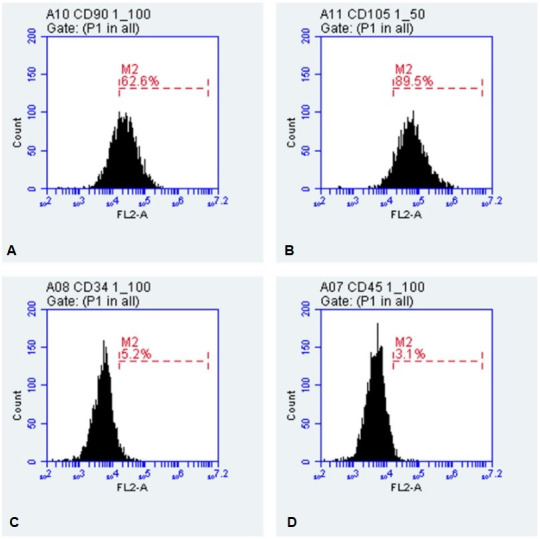
Stem cells membrane markers analysis. In (A) the CD90 and in (B) CD105 strains responded positively to the marking. The hematopoietic markers in (C) CD34 and in (D) CD45 showed negative labelling.

The flow cytometry analysis showed that mesenchymal cells derived from MAC, showed positive staining for CD90 (62.6%) and CD105 (89.5%). The negative staining was restricted to hematopoietic markers CD34 (5.2%) and CD45 (3.1%) (Figure 6).

### Small extracellular vesicles are affected by cell passage

**Figure 7:**
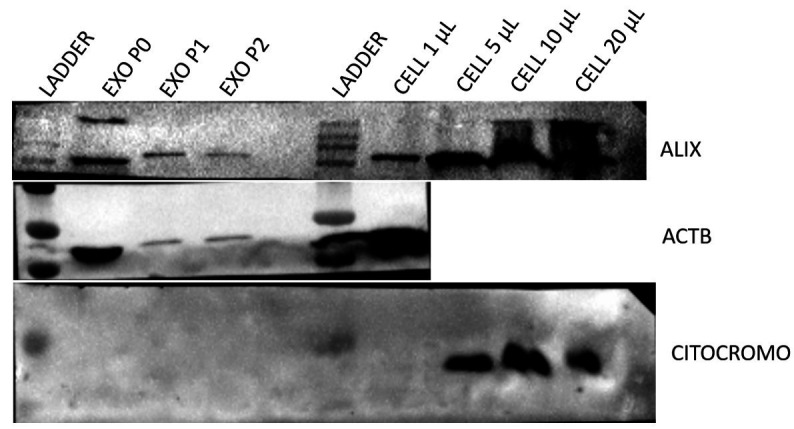
Small extracellular vesicles protein analysis. Observe positive marking for Alix and ACTB in exosomes derived from MAC. Cytochrome showed positive staining only for cells in different concentrations.

**Figure 8:**
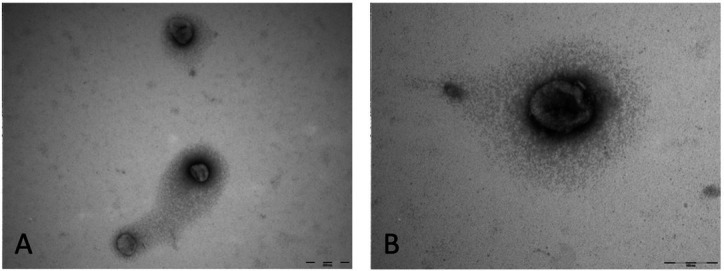
Transmission electron microscopy analysis of EVs obtained of conditioned medium by canine amniotic membrane stem cells. The magnification of the picture was (A) 100,000x and (B) 200,000x.

**Figure 9:**
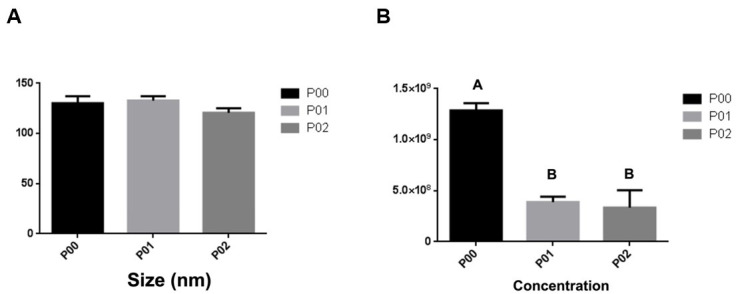
Nanoparticle tracking analysis. In (A) Observe that in all measured passages (P0 to P2) the size of the EVs derived from MSC has the same pattern. In (B) Observe that the cells in P0 showed a much greater secretion effect of small EVs when compared to the same cells in P1 and P2. Standard bars represent the standard error of the mean (SEM). Different letters indicate (p<0.05).

The culture medium samples went through processes followed by centrifugation, as previously described. The samples remained in the freezer at -80 °C until thawing for the ultracentrifugation process, Western Blotting, TEM and nanoparticle tracking analyses. A total of nine samples of stem cells from the canine amniotic membrane were analyzed in passages P0, P1 and P2. Western Blotting analysis for small extracellular vesicles specific antibodies, demonstrated the presence of ALIX and ACTB proteins, demonstrating that the particles isolated from the canine amniotic membrane stem cell culture are small extracellular vesicles. MSC cells also underwent analysis and were positive for Cytochrome, demonstrating the lack of contamination in the EVs samples (Figure 7). In order to evaluate the small extracellular vesicles separation and morphology we used transmission electron microscopy. The images demonstrated the separation of cup-shape vesicles with sizes in average around 100nm and smaller (Figure 8 A and B). Additionally, the images demonstrate the lack of co-precipitants such as protein aggregates. The particles observed by nanoparticle tracking had the size expected for small EVs in all three cell culture media analyzed (P0 to P2), with P0 = 132nm, P1 = 130nm and P2 = 120nm (Figure 9). The concentration of EVs was higher in P0 compared to passages P1 (p<0.003) and P2 (p<0.002) as demonstrated in Figure 9. Thus, the results demonstrate that the establishment of the cell culture affect EVs concentration throughout the culture period demonstrated by the decrease in total number of particles isolated from the media.

## Discussion

The use of mesenchymal stem cells from adult tissues for research purposes is being widely used due to their potential for proliferation. With the increase in age and the appearance of congenital diseases in dogs, the study of these cells growing and consequently the understanding about their biology and function will allow the elucidation of questions related to these cells. (Russell et al., 2016). MAC are the source for this project since it is a tissue that is part of the fetal attachment, and is discarded as biological waste after pregnancy delivery, therefore its collection is not invasive, which makes the collection of this tissue as an ethically accepted source of MSC. Based on that, in this manuscript the MAC collection was performed under sterile conditions, incising the uterine horns with a scalpel blade until the fetuses were exposed. Upon collection cells were plated and evaluated for cell differentiation. Cell culture media was collected in passage P0, P1 and P2 for small extracellular vesicles analysis.

The MAC were mechanically separated from the chorion and rinsed with 5% penicillin/streptomycin solution. Then it was macerated with scalpel blades until it was a homogeneous mass. Chemical digestion was started to complete the first stage. After chemical digestion, the cells were plated together with basal culture medium where they remained until the replication processes. The protocols adapted from Park et al. (2012); Vidane et al. (2014) and Cardoso et al. (2017) used to extract the cells in this work, proved to be efficient. However, obtaining the tissue is not always uniform and constant, due to the dependence on the availability of pregnant bitches. MAC cells were proliferative and rapidly growing, in fibroblastoid format, corroborating the results of Lange-Consiglio et al. (2012) and in horses, Park et al. (2012) and Saulnier et al. (2016) in MAC and Vidane et al. (2014) in feline amniotic membrane, which showed that cultures of amniotic membrane presented cells with a fibroblastoid format. Fibroblastoid morphology and adherence to plastic, observed throughout the culture of MAC cells, is a characteristic of MSCs, as previously described by Dominici et al. (2006); Filioli Uranio et al. (2011); Park et al. (2012); Saulnier et al. (2016); Cardoso et al. (2017) and Borghesi et al. (2017). The cells were tested after the cryopreservation process to prove cell viability. Due to the large percentage of living cells (approximately 90%), we were able cryopreserved these cells to be used in future research. Similar capacity for cell viability was previously described by Lange-Consiglio et al. (2012) and Park et al. (2012) in MAC cells and Borghesi et al. (2017) in rabbit amniotic membrane cells. The growth curve was analyzed and a rapid cell growth was observed during the first passages (P0 to P2), with its peak growth in P2 and subsequent decline in the next passages. These results corroborate the findings of Vidane et al. (2014) in the use of the same cells as cats and Filioli Urânio et al. (2011) in dogs. However, our results were different when compared to Park et al. (2012), which used the same type of canine cells, and reported constant growth until the twentieth passage. In Double time analysis, MAC cells showed an increase in the number of days for cell duplication, reaching senescence in the fifth pass, when it was no longer viable, that is, as the number of cultivation passages increased, cells lost their ability to proliferate, sharing the same results as Filioli Urânio et al. (2011) in dogs and Vidane et al. (2014) in cats. The evaluation of colony formation capacity (CFU) was carried out, showing a large amount of CFU and with varying number of cells, which favors its application in clinical therapies (Nardi and Meirelles, 2006).

MAC cells were tested for their ability to differentiate into adipogenic, chondrogenic and osteogenic lineages, which is also one of the tests used to classify them in MSCs (Dominici et al., 2006; Filioli Uranio et al., 2011; Wenceslau et al., 2011; Park et al., 2012; Saulnier et al., 2016; Cardoso et al., 2017; Borghesi et al., 2017). In the flow cytometry analysis, the cells showed positive marking for mesenchymal markers (CD90 and CD105) and did not express the hematopoietic markers (CD45 and CD34), thus confirming one of the factors imposed by Dominici et al. (2006) to characterize a stem cells as mesenchymal. Our results corroborate with other studies using MAC (Lange-Consiglio et al., 2012; Park et al., 2012; Cardoso et al., 2017), in rabbits (Borghesi et al., 2017) and on human amniotic membrane Yamahara et al. (2014), however with feline amniotic membrane Vidane et al. (2014) reported low expression for CD105 markers.

In this study, in addition to the characterization of stem cells from the canine amniotic membrane, the size and concentration of the extracellular vesicles secreted from these cells were also evaluated. The isolation protocol proved to be efficient, having the EVs analyzed by nanoparticle tracking analysis, which demonstrated the expected size (up to 150nm) and morphology according to TEM. The same success was achieved with this protocol in the work of Connolly et al. (2015) when extracting EVs from human adipocytes and with Zhao et al. (2017) who in their work isolating EVs from human amniotic membrane. Additionally, the presence of protein markers was evaluated by western blot and demonstrated that the separated EVs are indeed small extracellular vesicles and lack cell contamination. In the characterization by nanoparticle tracking analysis it was possible to notice that the EVs in the different analyzed passages (P0 to P2), did not demonstrated significant differences in their sizes when compared among themselves (P0 = 132nm, P1 = 130nm and P2 = 120nm). Similarly, in the work by Connolly et al. (2015), with EVs derived from human adipocytes, which compared the secretion of EVs by cells on day zero and on day fifteen of culture, EVs did not presented differences in size along the culture period. The EVs size determined at different stages of culture suggests that they are small extracellular vesicles, as they have the expected size of up to 250nm by nanosight and 200nm by TEM (Zhang et al., 2015; Théry et al., 2018). When analyzing the concentration, also by Nanosight it was possible to observe that the EVs presented a higher concentration in the P0 passage when compared to P1 and P2. The same result was possible to be observed by Connolly et al., (2015) who analyzed the concentration of EVs derived from human adipocytes which demonstrated that the production of EVs per cell was higher on day zero of culture when compared to day fifteen. The fact that EVs production is higher in the early stages may be due to the role of EVs as intercellular communicators and that with the arrival of the senescence process, that is, when their ability to multiply comes to an end, the production capacity of EVs also decreases. It can be suggested that the cellular passage process significantly affects the intercellular communication capacity, which may be an additional factor playing a role to a sudden drop in the growth curve after the second passage (P2).

## Conclusion

With this work, we concluded that a greater number of small extracellular vesicles are present in the passage zero when compared to passages one and two, suggesting that communication in this phase is greater, that is, this would be the best phase for application in pre-clinical tests. However, additional studies are necessary to verify the contents of this EVs as well as the cell response upon treatment.
